# A repurposing screen of 29 media mixtures derived from 15 commercial serum-free media products identifies potential cost reductions for industrial animal cell cultivation

**DOI:** 10.1007/s13205-026-05091-5

**Published:** 2026-10-15

**Authors:** Matthew A. J. Wasmuth, Darren N. Nesbeth

**Affiliations:** Department of Biochemical Engineering, University College London, Bernard Katz Building, London, WC1E 7JE UK

**Keywords:** Serum, Animal cell cultivation, Repurposing screen, Cultivated meat

## Abstract

We performed a repurposing screen of 15 commercial serum-free products, across 29 different media mix options, in Nunc™ EasYFlask™ cell culture flasks/CELLBIND® 6-well plates, agnostic of their recommended application or cell type. 20% v/v FBS DMEM was replaced with a given serum-free mix in a single step, and growth monitored over 6 days for the mouse muscle cell line, Sol8. 20 serum-free media mixes reduced or failed to increase growth, while 9 increased cell growth. 'Mix D', Fishers base medium containing 2% v/v Ultroser G serum alternative, showed the best combination of cell number increase and volumetric media cost reduction: 33% lower than 20% v/v FBS DMEM. Further Nunc™ EasYFlask™ cell culture flasks/CELLBIND® 6-well plates experiments showed that Mix D supported levels of Sol8 cell growth comparable to 20% v/v FBS DMEM, but with lower levels of metabolic activity. Comparable growth in Mix D was observed for Sol8 cells that had undergone a period of adaptation to the serum-free medium, and clonally-derived Sol8 sub-populations. Mix D also matched serum-containing media performance in supporting growth of the porcine muscle cell line, pMYOSC, the porcine pre-adipocyte fat cell line, pADSC and the mouse pre-adipocyte cell line, 3T3 L1 in Nunc™ EasYFlask™ cell culture flasks/CELLBIND® 6-well plates. 3T3 L1 cells cultivated in Mix D retained their capacity to differentiate into adipocytes, a cell type important for animal fat cultivation. The observation that Mix D supported growth of four different animal cell types at levels comparable to serum-containing medium recommends this formulation, and this repurposing screen approach, to researchers seeking serum alternatives.

## Introduction

Foetal bovine serum (FBS) remains one of the most widely used supplements in animal cell culture, owing to its rich content of growth factors, hormones, and adhesion molecules that support proliferation and viability across a wide range of cell types (Brunner et al. 2010). However, its use poses significant industrial challenges. FBS remains one of the most expensive and controversial cell culture supplements, due to its reliance on bovine fetuses sourced from the slaughter industry (Brunner et al. 2010; Gstraunthaler et al. 2013). Batch to-batch variability may undermine reproducibility (van der Valk et al. 2018), while its animal origin raises potential ethical and biosafety risks (Gstraunthaler et al. 2013; European Food Safety Authority 2024) and religious concerns (Mazlan et al. 2025). Moreover, the high and volatile cost of FBS is a major cost consideration for large-scale applications such as cultivated meat production and regenerative medicine manufacturing (Beheshtizadeh et al. 2022; Quek et al. 2024; Oey et al. 2026).

### Growth media

Modern tissue culture media comprise two principal components: base media, which supply fundamental nutrients, buffers, and salts; and supplementary factors, traditionally derived from animal serum but increasingly replaced by defined or chemically characterized serum alternatives. Both base media and serum replacement components tend to be commercial secrets, although some general features are common knowledge.

Base media formulations trace their lineage to the pioneering work of Morgan et al. (1950), Dulbecco and Vogt (1954) and Eagle (1955) in establishing minimal essential medium (MEM) by systematically determining the nutritional requirements of mammalian cells in culture in settings such as cancer and virology research. Animal cell base media formulations typically provide carbohydrates for energy metabolism, amino acids for protein synthesis, vitamins as enzymatic cofactors, inorganic salts for osmotic regulation, and buffering systems to maintain physiological pH.

FBS provides a complex mixture of growth factors, hormones, attachment factors, lipids, carrier proteins, and trace elements necessary for optimal cell performance. Contemporary serum alternatives cover a spectrum of compositional definitions, from serum-free media containing undefined animal-derived components such as hydrolysates and lipid fractions, to chemically defined formulations comprising exclusively characterized molecular entities, to protein-free media that eliminate all protein-based supplements. These formulations typically incorporate recombinant or purified growth factors (insulin, transferrin, epidermal growth factor, fibroblast growth factors), lipid carriers (albumin or synthetic alternatives), attachment factors (fibronectin, vitronectin, or synthetic peptides), antioxidants, lipids and fatty acids, trace elements, and synthetic polymers such as polyvinyl alcohol or Pluronic F-68 (Rodeheaver et al. 1980) to provide shear protection and surface activity. So-called 'complete' serum free media products provide both the base media and serum functions.

### Serum replacement

For cultivated meat, current estimates (Liu et al. 2026; Sundaram et al. 2026; Xie et al. 2026) suggest that growth media account for approximately 55–99% of total cultivated meat production costs, with FBS itself contributing around 60–80% of total media expenditure. Consequently, considerable effort has focused on replacing serum with chemically defined media in which serum functions are reconstructed using defined combinations of insulin, transferrin, selenium, recombinant growth factors and functional proteins. However, these replacements can themselves dominate media costs through reliance on expensive recombinant proteins such as Fibroblast Growth Factor 2 (FGF2), Transforming growth factor-beta (TGF-β) and albumin (Quek et al. 2024).

To reduce the economic burden of serum supplementation, numerous alternative serum replacement strategies have been reported. Interested readers should consult excellent reviews by Oey et al. (2026) and Sundaram et al. (2026) for discussion of how plant protein hydrolysates, rapeseed protein, soybean-derived supplements, microbial extracts, yeast products, microalgal biomass and lysates, insect-derived proteins, milk whey and agricultural side-streams have been investigated as components of serum replacement technologies.

More sophisticated approaches that have been explored include recombinant proteins produced in microbial or plant expression systems, genetically engineered autocrine cell lines and media recycling approaches, with microalgal co-culture and lysate supplementation emerging as particularly promising multifunctional solutions capable of simultaneously reducing serum, amino acid and media replacement requirements (Quek et al. 2024).

A growing number of serum-free and serum-replacement products have entered the market, claiming to support equivalent or improved cell growth while reducing cost and ethical burden. Comparative analyses of these products remain sparse, often focusing on a narrow subset of cell types or lacking head-to-head benchmarking under identical conditions (Gstraunthaler 2003). Furthermore, while some studies have reported partial success in replacing FBS (Rasor et al. 2021), systematic, agnostic screens are needed to identify affordable alternatives that are robust across multiple cell types.

The selection of serum alternatives for animal cell cultivation is traditionally guided by manufacturer specifications that categorize products according to intended application scenarios—most commonly for biopharmaceutical production (Rodrigues et al. 2012) and regenerative medicine (Yang et al. 2025). However, this application-centric approach may inadvertently constrain optimization efforts and overlook superior performing formulations that fall outside conventional categorizations. An agnostic selection strategy, whereby serum alternatives are evaluated based solely on their performance and compatibility with specific cell lines rather than their marketed applications may potentially offer advantages for advancing cell cultivation science.

Manufacturer technical documentation necessarily reflects commercial positioning strategies that may not align with the realities of cellular metabolism and growth kinetics. Products developed and marketed for biopharmaceutical applications, for instance, are often positioned based on regulatory considerations and established precedents rather than fundamental performance superiority. Conversely, formulations developed for research-grade applications may exhibit equivalent or superior performance characteristics yet remain unexplored for manufacturing purposes due to their commercial classification. This artificial segregation of serum alternatives into application-specific categories potentially creates silos that could inhibit systematic comparison across the full landscape of available formulations. Furthermore, the metabolic requirements of mammalian cells in culture—encompassing growth factors, hormones, attachment factors, lipids, and trace elements—are fundamentally determined by cellular physiology rather than by end-use application.

### This study

In this study we approached serum alternative selection through an agnostic lens that prioritized direct empirical assessment of growth performance and cost, in a manner analogous to the use of repurposing screens to identify novel disease models (Mertens et al. 2023) or drug functions (Sake et al. 2024). Given that industrial animal cell cultivation must simultaneously satisfy constraints of cost, scale, and robustness to non-adapted medium transitions that are largely absent from biopharmaceutical or basic research contexts, empirical screening approaches that test products outside their marketed application are particularly well suited to this setting, where manufacturer categorisation is least likely to reflect performance under industrially relevant conditions. This evidence-based methodology has the potential to accelerate process development timelines and reduce development costs, and ultimately identify superior cultivation conditions.

We selected 15 commercial serum alternative products (Table 1), identified from the ‘FCS-free’ online database provided by Utrecht University (3RCU 2024) for inclusion in a repurposing screen using the murine Sol8 myoblast cell line. Sol8 myoblasts can develop and mature into multinucleated myotube networks (Marchand et al 2001) and have been used as a model of normal muscle function for in vitro investigation of congenital muscle disease (Hill et al. 2004), muscle development (Calera and Pilch 1998), and the efficacy of food supplements in improving muscle health in exercise recovery (Xu et al. 2025). The status of mouse myoblasts as a model for muscle tissue developments also lends them relevance to emerging bioindustrial sectors such as cultivated meat (Tuomisto et al. 2022; Dos Santos et al. 2024), where mouse cells have recently been approved as a pet food ingredient (Cleaver 2025), and animal testing alternatives (Kane et al. 2009; Jhuang et al. 2025).

**Table 1:** Cost profiles of serum-free media mixes used in this study

	1																3 Serum Cost %
	Complete Serum-Free Media					Base Media						Serum Alternatives				FBS	
	BHK21	M199	IMDM	Hams F12	AIM-V	Fishers	DMEM	Swims	Ames	Williams	Iscoves	Ultroser G	AOF-ITS	Knockout	B27		
Component £/ L	£3.92	£8.88	£17.05	£93.40	£104	£6.00	£37.32	£6.02	£6.68	£6.68	£6.68						
Component £/ mL												£4.598	£4.22	£0.526	£9.24	£0.616	

2																		
Mix code	Mix £/L	Volume (mL) of component used in 1L																3 Serum Cost %
A	£48.14						990							10				88
B	£79.15							990						10				53
C	£135.06							800							200			78
D	£97.83						980						20					94
E	£128.52							980					20					72
F	£110.00						800								200			96
G	£3.92	1000																
H	£8.88		1000															
I	£17.05			1000														
J	£97.85								980				20					94
K	£98.50											980	20					93
L	£48.81											990		10				86
M	£110.54											800			200			95
N	£93.40				1000													
O	£48.16								990					10				88
P	£110.02								800						200			96
Q	£98.50									980			20					93
R	£48.81									990				10				86
S	£110.54									800					200			95
T	£98.50										980		20					93
U	£48.81										990			10				86
V	£110.54										800				200			95
W	£221.37							980								20		83
X	£286.72						980									20		64
Y	£191.35											980				20		97
Z	£190.70								980							20		97
AA	£191.35										980					20		97
AB	£191.35									980						20		97
AC	£104.00					1000												
20	£153.06							800									200	80

Section 1 (indicated on the leftmost column) details the 15 component names, along with their 2024 cost per L, or mL for clarity. Section 2 details the recipe for 1 L of each 'mix' investigated in this study, in terms of mL of each component used and the volumetric cost of each mix per L. The rightmost column (Sect. 3) indicates the percentage of the cost of each mix which is required for the serum alternative

**Fig. 1:**
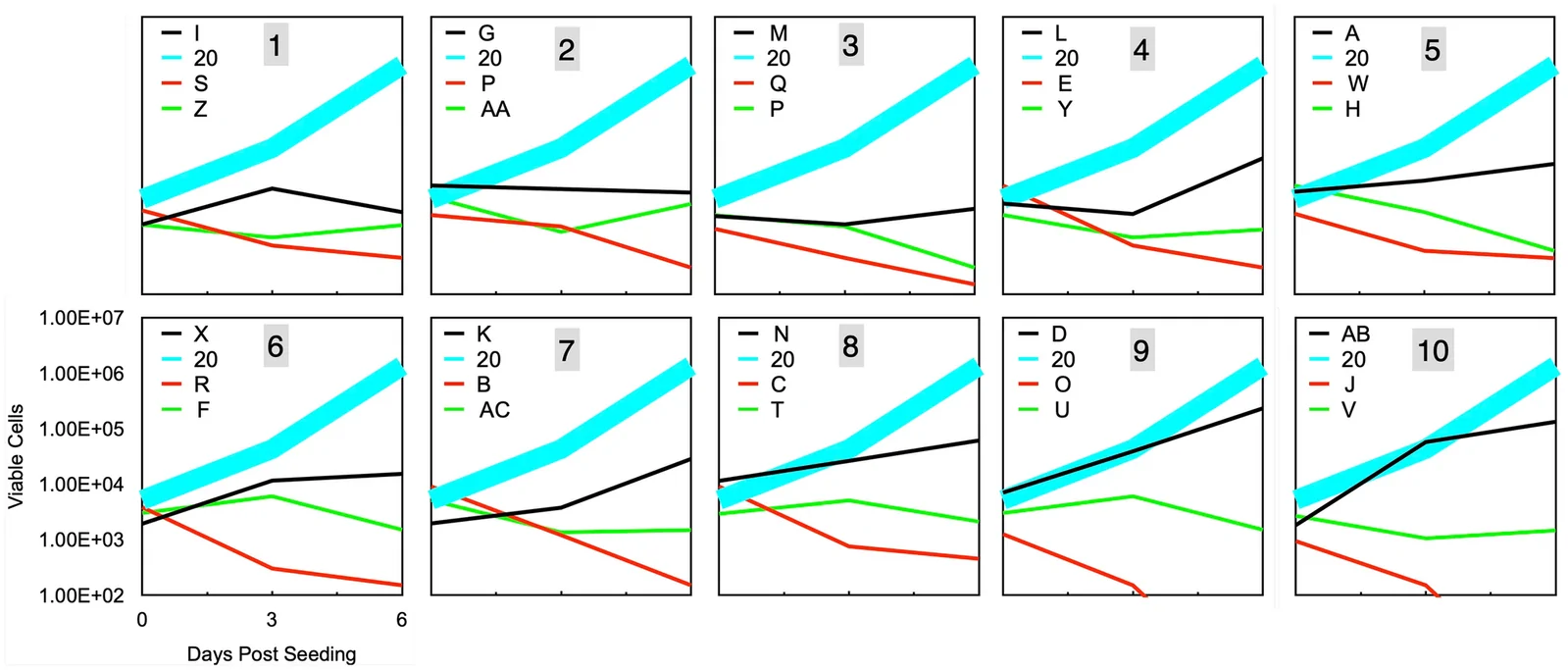
Sol8 cell growth over 6 days in 29 different media mixes. Ten Sol8 cell growth plots (1–10), each featuring growth in up to three different exprimental media mixes (red, black and green) compared compared to growth in in 20% v/v FCS DMEM (light blue). For 29 different experimental media mixes, Sol8 cells harvested from cultivation in 20% FCS DMEM were harvested and counted and then 2 × 10^3^ cells resuspended in 2 mL of a given media mix, including for 20% FCS DMEM and transferred to 6 well plate wells. The initial cell count was logged as a 'Day Zero' count. Cells from one 6-well plate well were harvested and counted 3 days post-seeding and from the second 6-well plate well 6 days post seeding. Viable Sol8 cell growth performance for all media types was plotted in groups of up to four data profiles across ten plots (1–10). Growth in 20% FCS DMEM was plotted in blue and the data re-plotted across all ten plots. For plots 2–10 growth in different media mixes within a given plot was colour-coded as either black (highest day 6 cell count), green (next highest day 6 cell count) or red (lowest day 6 cell count). Plot 1 omits a black-coded data profile. The identity of each media mix is indicated by the key within each plot, using the lettering code from Table 1, with '20' indicating 20% v/v FCS DMEM. All ten plots have the same axes range and labelling as plot 6. For each media mix, on days 3 and 6 post-seeding, a single cell count was performed for cells harvested from a single 6 well plate well

**Fig. 2:**
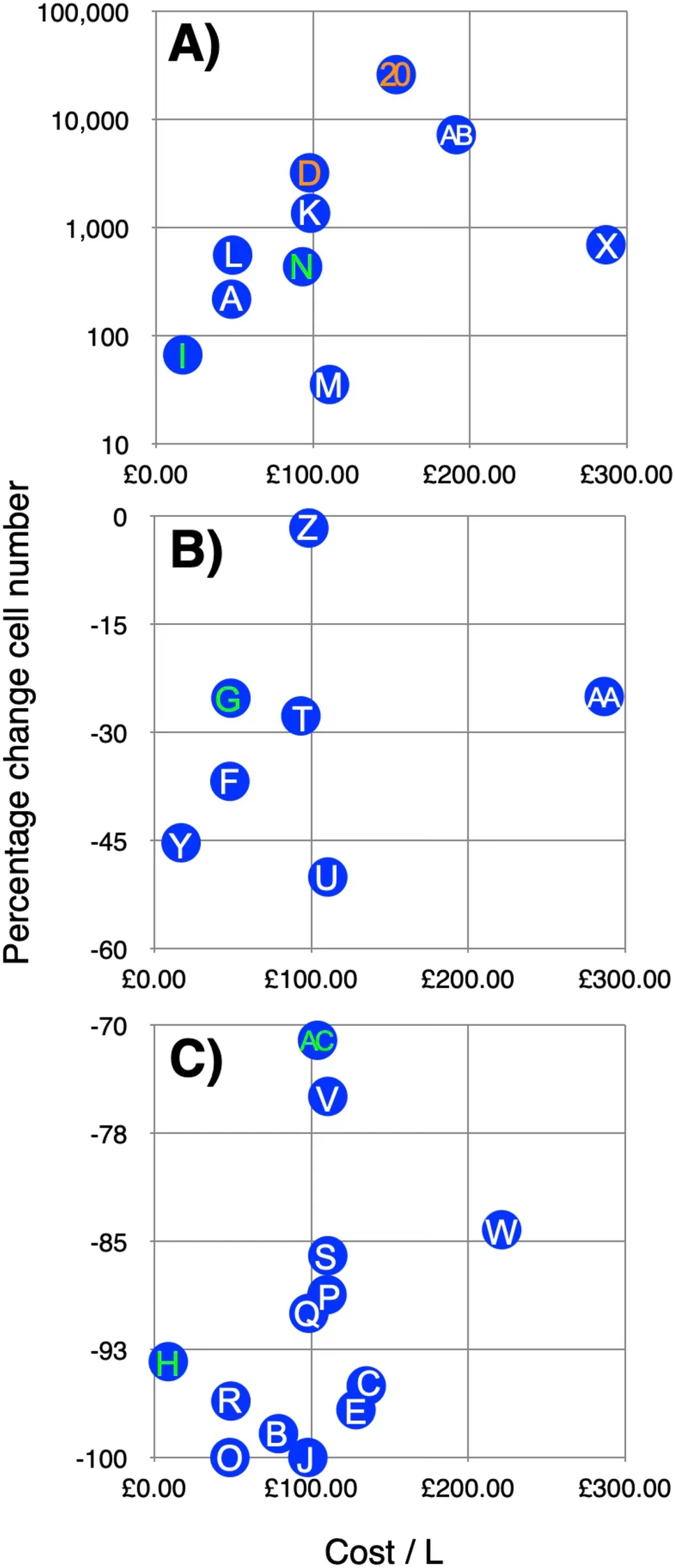
Sol8 cell growth performance as a function of volumetric cost for 29 media mixes. Three plots (**A**–**C**), indicating growth data from Fig. 1 express as percentage change in cell number, as a function of media mix cost per litre. The different Y axis ranges of each plot allow resolution of each data point. For each of the media mixes tested in Fig. 1, the percentage increase in total viable cell number from Day zero to Day 6 was calculated. To aid visualisation data points were distributed across plot (**A**), with a logarithmic Y axis for media where growth increased, and linear axes used for plot (**B**) for media that resulted in a cell number unchanged or reduced by up to 60% on Day 6, and plot (**C**) for media that resulted in a reduction of between 70 and 100%. Identity of each media mix is indicated as in Fig. 1. Orange font colour highlights Mix D and 20% v/v FCS DMEM. Green font colour highlights those media mixes comprised only of 'complete' media products (Table 1)

**Fig. 3:**
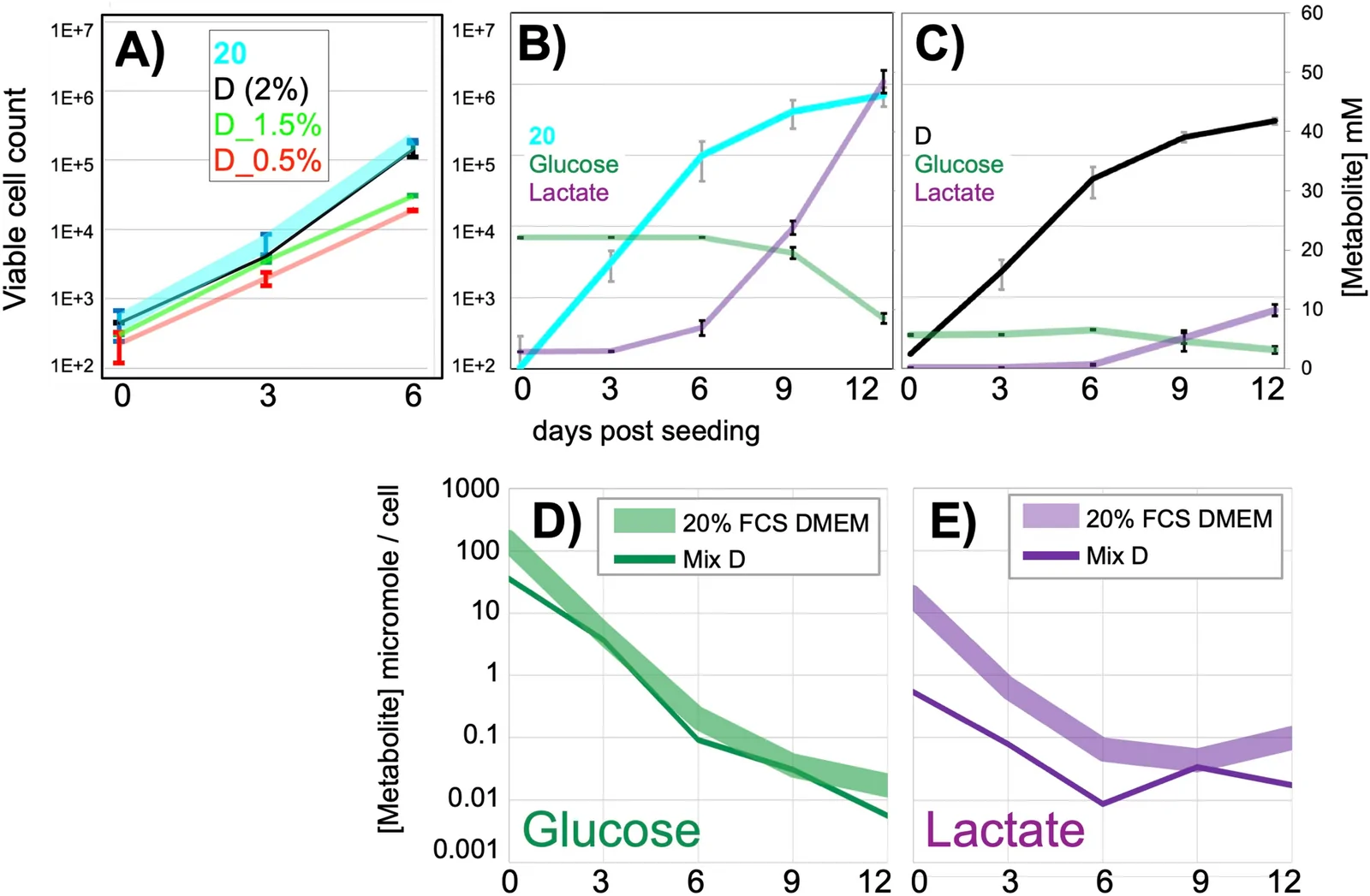
Ultroser G concentration effects in Mix D and Sol8 cell metabolic activity. **A** Sol8 cell growth plot featuring growth in up to three different experimental media mixes (red, black and green) compared compared to growth in in 20% v/v FCS DMEM (light blue). Sol8 cells were harvested and seeded as described in Fig. 1, but at a total of 2 × 10^2^ cells in 2 mL to a 6 well plate well 20% v/v FCS DMEM (blue data profile), Mix D with Ultroser G concentration at its standard 2% v/v level (black data profile labelled **D**), or reduced to 1.5% v/v (green data profile labelled D_1.5%) or 0.5% v/v (red data profile labelled D_0.5%). N = 3 seedings were performed for each media type, 3 seeded wells were harvested for a given timepoint and single cell count performed. **B** Sol8 cell growth in 20% v/v FCS DMEM (blue data profile), and levels of glucose (green data profile) and lactate (purple data profile) up to 12 days post-seeding. **C** As in panel B but growth media used was Mix D (black data profile). For panels (**A**–**C**), N = 3 seedings were performed for each media type, 3 seeded wells were harvested for a given timepoint, pelleted by centrifugation and then cells were counted via n = 3 repeat counts, and each of glucose and lactate determined over n = 3 repeat measurements. Data points are mean values, and error bars are standard error. **D** Glucose and cell cound data from panels (**B**, and **C**) were used to plot micromoles glucose per cell at indicated days post seeding for cells grown in 20% v/v FCS DMEM (thick green data profile) and Mix D (thin green data profile). **E** As panel (**D**) except micromoles lactate per cell, for cells grown in 20% v/v FCS DMEM (thick purple data profile) and Mix D (thin purple data profile)

**Fig. 4:**
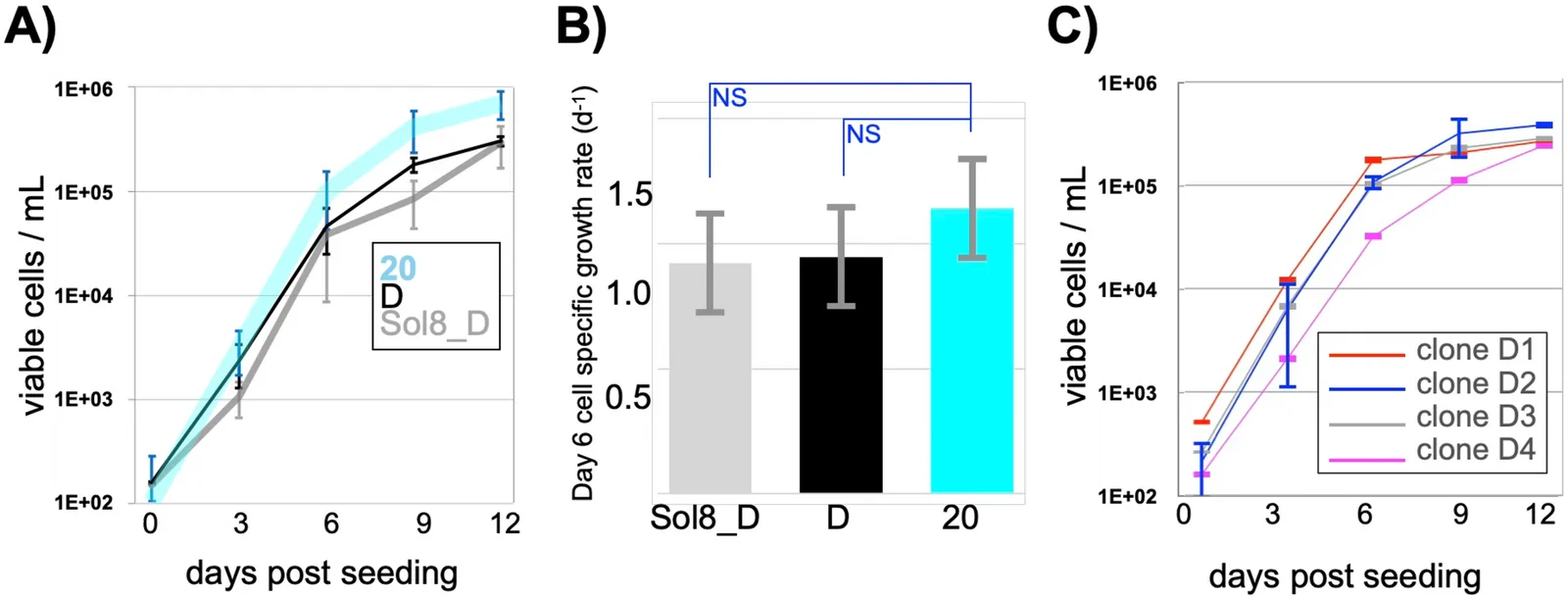
Growth in Mix D of both adapted and clonally derived Sol8 populations. **A** Sol8 cells were seeded into a 6-well plate as in Fig. 1, but at a total of 2 × 10^2^ cells in 2 mL, using 20% FCS DMEM (blue data profile, '20') or Mix D (black data profile 'D'), and cultivated over 3 passages, with cell counting performed as part of each passage procedure. After three passages in Mix D, the endpoint cells were considered as having been adapted and were labelled as 'Sol8_D'. These Sol8_D cells were then subsequently seeded and cultivated in Mix D in the same manner as before, and the data also plotted onto this graph (grey data profile 'Sol8_D'). **B** Cell Specific Growth Rates (CSGR) were derived from the data in plot A). Mean values of N = 3 determinations were plotted as data bars, with error bars being the standard error. Growth rates were compared by two-tailed Student's t-test with a Bonferroni-corrected significance threshold, using 20% FCS DMEM ('20', blue) as the reference condition: '20' vs. Mix D ('D', black), and '20' vs. Mix D with adapted cells ('Sol8_D', grey). Both planned comparisons used a corrected threshold of α = 0.025 (two comparisons; 0.05/2); neither reached significance (p = 0.998 and p = 0.607, respectively), indicating no statistically significant (NS) difference in growth rate between the reference condition and either alternative**. C** Four clonally derived subpopulations (clones D1-D4) of Sol8_D cells were isolated by limiting dilution cloning in Mix D. Cells of each were then harvested and seeded in Mix D and cell counts taken up to 12 days post seeding. For panels (**A** and **C**), triplicate seedings were performed for each media type, and cell counts performed in triplicate for each seeding. Data points are mean values, and error bars are standard error

**Fig. 5:**
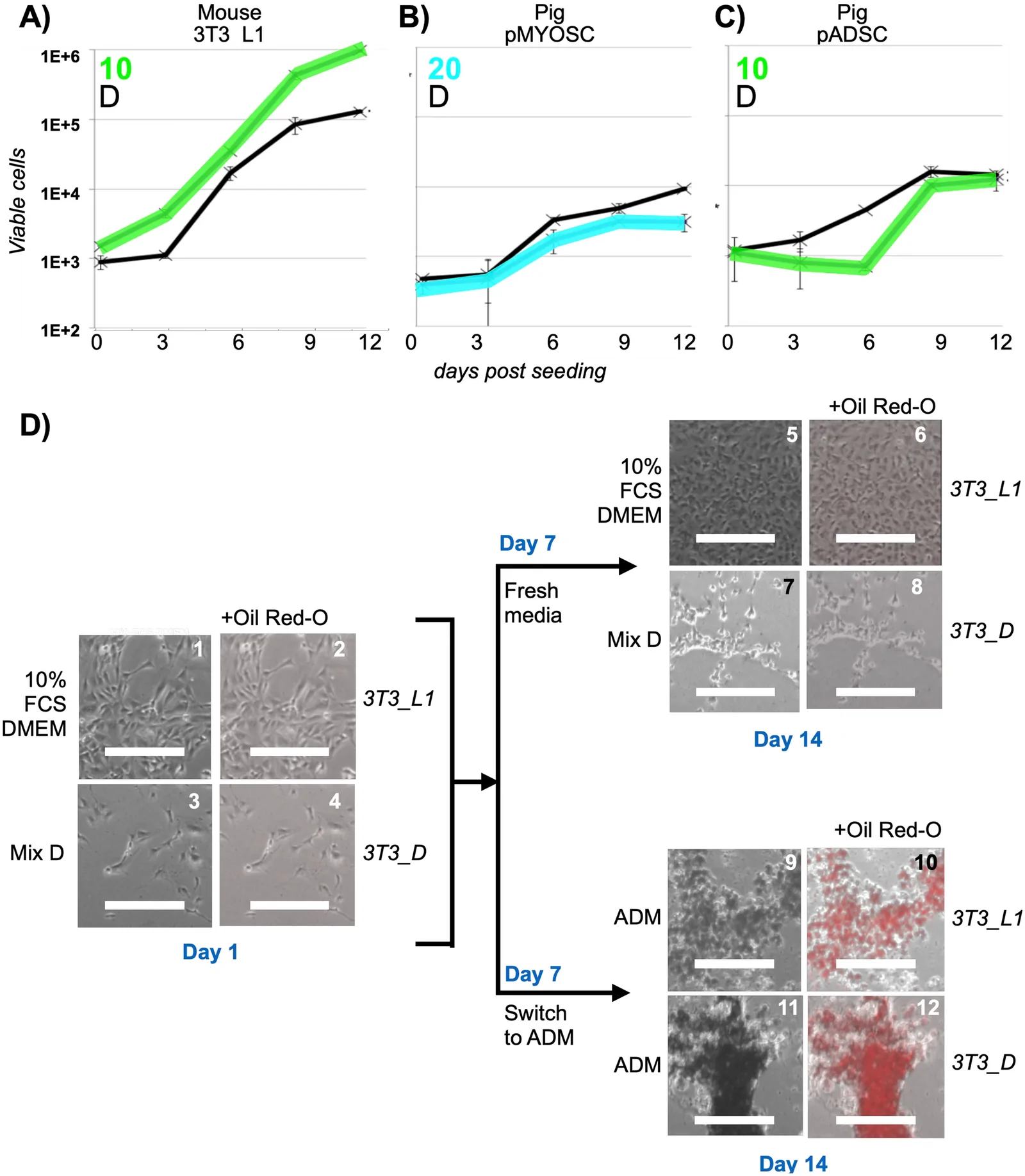
Mix D supports growth of 3T3, pMYOSC and pADSC cells growth plus adipocyte differentiation of 3T3 cells. **A** N = 3 seedings of 2 × 10^3^ mouse 3T3_L1 cells into wells of a 6-well plate were performed and three seeded wells were harvested for a given timepoint, and single cell counts performed for each. Growth media used was 10% v/v FCS DMEM (green data profile labelled '10') or Mix D (black data profile labelled 'D'). Both data profiles are representative. **B** As panel A), but pMYOSC cells were cultivated in 20% FCS (blue data profile labelled '20') or Mix D (black data profile labelled 'D'). **C** As panel A), but pADSC cells were cultivated in 10% v/v FCS (green data profile labelled '10') or Mix D (black data profile labelled 'D'). Data profiles are representative for serum-containing media in panels B) and C). For Mix D in plots B) and C), data points are mean values, with error bars being the standard error. **D** 3T3_L1 cells were seeded into wells of a 6-well plate, but at a total of 2 × 10^4^ cells in 2 mL media, in 10% FCS DMEM. 1 day post seeding ('Day 1' label in blue), microscopy images (panels 1–2) were captured immediately before addition of Oil Red-O (no label above images), and 30 min after (label '+Oil Red-O' above image). The cell line '3T3_D' was generated by adapting 3T3_L1 cells to grow in Mix D, with the same procedure used to generate the Sol8_D cell line in Fig. 4. 3T3_D cells were seeded into Mix D in the same way as 3T3_L1 cells, with equivalent images taken 1-day post-seeding (panels 3–4). 7 days post-seeding the media for both cell types was removed and replaced with either a fresh volume of the same media type (upper connector arrow labelled 'Fresh media') or the ADM differentiation media (lower connector arrow, labelled 'Switch to ADM'). 21 days post seeding, images were again captured before (panels 5, 7, 9 and 11) and after (panels 6, 8, 10 and 12) Oil Red-O addition. All scale bars indicate 1 mm

We first identified formulations supporting high growth rates relative to FBS (Fig. 1), then assessed their performance relative to cost (Fig. 2). We captured metabolic activity data (Fig. 3), investigated whether media adaptation could enhance growth in the leading media mix (Fig. 4), and evaluated cross-lineage compatibility across further murine and porcine cell types (Fig. 5). With this integrated approach we aimed to identify serum alternatives that balance biological performance and economic feasibility, thereby advancing sustainable mammalian cell culture practices.

## Methods

### Cell provenance and cultivation

All cell culture was performed in a Hera Cell 150 incubator (ThermoFisher Scientific, UK) set at 37 °C and 5% CO_2_ using either Nunc™ EasYFlask™ cell culture flasks (ThermoScientific, USA) or CELLBIND® 6-well plates (Corning, USA) on shelves in static conditions. All cultivation was performed using complete StemPro™ Adipogenic Differentiation media (ThermoFisher Scientific, USA) or media detailed in Tables 1 and 2. All media were supplemented with Antibiotic–Antimycotic™ (ThermoFisher Scientific, USA) to a concentration of 1% v/v. Cell counts and viability were determined using a NucleoCounter NC-3000 (Chemometec, Denmark) and 200 μL aliquot samples within compatible Chemometec Via1-cassette vessels. Sol8 cells were purchased from ATCC (CRL-2174., USA), 3T3 L1 cells were purchased from ATCC (CL-173., USA). pADSC cells and pMYOSC cells were a kind gift from Paul Cameron, UCL Biochemical Engineering. Briefly, the pMYOSC cells and pADSC were derived from piglet muscle tissue or adipose tissue respectively by staff at the Royal Veterinary College, London, UK, using ethically approved procedures and had subsequently undergone phenotype confirmation and three passages in 20% v/v FBS DMEM prior to their use in this study.

**Table 2:** Serum-free media products used in this study

Name	Catalogue number	Supplier
IMDM	12,440,053	Thermofisher Scientific, USA
DMEM	10,569,010	Thermofisher Scientific, USA
CD-BHK-21	A1627703	Thermofisher Scientific, USA
M199	51322C	Merck, USA
DMEM-Hams-F12	D9785	Merck, USA
AIM™-V	12,055,083	Thermofisher Scientific, USA
Fischers	21,475,025	Thermofisher Scientific, USA
Swim’s	MBS653080	MyBioSource, USA
Iscove’s	MBS653378	MyBioSource, USA
Ultroser™ G	15,950–017	Sartorius, Germany
AOF-ITS	SCM054	Merck, USA
KnockOut™	10,828,028	Thermofisher Scientific, USA
B-27™	17,504,044	Thermofisher Scientific, USA
FCS	10,082,147	Thermofisher Scientific, USA

Catalogue number and supplier, location indicated

### Cell growth rate calculations

Cell count values were used to calculate cell specific growth rate, *μ*, in units of day^−1^ (d^−1^) using Eq. 1.

1 $$ \mu = \frac{{Ln\left( {C_{t} /C_{t0} } \right)}}{t - t0} $$

**Equation 1.** Determination of cell specific growth rate, *μ*. *C*_*t*_ = Cell number at measured time point, *C*_*0*_ = Cell number at seeding, *t* = measured time point, *t0* = cell seeding time point.

### Microscopy

Cells were imaged using an EVOS FL microscope (AMEX1000, ThermoFisher Scientific, UK) at 10X magnification. Phase-contrast were captured using the pre-set light cube settings in the EVOS FL system.

### Cell analytics

Lactate and glucose concentrations in cell culture media samples were determined using a CuBiAn Bioanalyser CuBiAn Bioanalyser (Optocell GmbH, Germany). Samples were centrifuged at 300 × g for 5 min, and the supernatant transferred to clean microcentrifuge tubes. Samples were stored at − 20 °C until analysis. For analysis, samples were thawed and diluted as required (typically 1:10 to 1:100 in deionized water depending on expected metabolite concentrations) to ensure readings fell within the linear range of the assay. Diluted samples (1 mL) were loaded into 1.5 mL Eppendorf’s and measured using appropriate calibration standards for lactate (0–20 mM) and glucose (0–30 mM). The Eppendorf’s were inserted into the CuBiAn Bioanalyser and metabolite concentrations determined using the manufacturer's proprietary enzymatic assay protocols with automated spectrophotometric detection. Results were automatically calculated from standard curves and corrected for dilution factors. All samples were analysed in duplicate and values reported as mean ± standard deviation.

## Results

We first identified 15 commercially available products designed to support serum-free cultivation of mammalian cells for use in our repurposing screen (Table 1). None of the products were, at time of selection, marketed for use in emerging biotechnologies sectors such as cultivated meat. Six base media products, four serum alternatives and five serum-free complete media products (Table 1, Sect. 1) were used as ingredients in 29 different media 'mixes' (Table 1, Sect. 2), each of which were arbitrarily assigned letter codes. Notably, the cost of the serum alternative element ranged from 53% (Mix B) to over 90% (for 14 different mixes) of the total, per volume, cost of a given mix.

### A binary repurposing screen identifies serum-free growth support without adaptation

To identify serum-free media mixes capable of supporting cell proliferation without requiring adaptation, we implemented a binary screening approach based on cell number changes over a 6-day culture period. Sol8 cells maintained in 20% v/v FBS DMEM were trypsinised, counted and 2 × 10^3^ cells re-seeded in a 2 mL of a given media mix in a single medium exchange, with no intermediate adaptation phase, and cell numbers were assessed at days 3 and 6 post-transfer.

We then enumerated the subsequent growth data (Fig. 1) on a low-replicate, binary pass/fail criterion for whether cell numbers did or did not increase. This provided a straightforward, high-throughput classification that enabled rapid elimination of inadequate mixes while identifying those warranting further investigation. We reasoned that media that could not support even modest proliferation over 6 days would be unlikely candidates for industrial animal cell cultivation application, regardless of other desirable properties they might possess.

The use of a 6-day time-frame was intended to allow potentially 3–4 population doublings, given many mammalian cell lines have doubling times of 24–48 h. 6 days would therefore likely provide sufficient time to distinguish between media that genuinely support cell proliferation versus those that merely maintain viability through depletion of intracellular nutrient reserves. We considered that the 6-day period would also balance the need for accurate biological characterisation against practical throughput considerations, as shorter periods risk false positives from cells subsisting on stored nutrients while longer periods potentially reduce screening capacity.

The absence of an adaptation step was intended to test whether the serum-free mixes would be robust to conditions representative of industrial scale-up scenarios where gradual medium transition is financially undesirable. Many bioprocess operations require direct medium switches during production campaigns, and transitions to serum-free media that necessitate lengthy adaptation periods can impose significant logistical and economic burdens. By screening under non-adapted conditions, we sought to prioritize identification of robust serum-free media mixes that could potentially function immediately upon implementation.

All 29 media mixes were screened and growth data colour coded according to highest (black), lowest (red) and intermediate (green) level of end-point growth (Fig. 1). With respect to this growth performance metric, the procedure identified mixes L, A, X, K N, D and AB as supporting Sol8 cell growth (Fig. 1, panels 4–10).

### Cost-performance analysis identifies promising serum-free media products

We next combined the cost information from Table 1 and end-point growth performance from Fig. 1 to plot two-dimensional data for comparison of all 29 serum-free mixes (Fig. 2). As cultivations were performed at small scale (2 mL working volume, 6-well plate format), the accuracy and biological meaningfulness of cell mass measurements would be limited. At this scale, biomass is typically below the reliable sensitivity threshold of standard mass-based quantification (e.g., wet/dry pellet weighing), where evaporative losses, residual media carryover, and pipetting variance introduce proportionally large errors. Cell number, by contrast, can be measured directly and reproducibly at this scale (e.g., by haemocytometer or automated counter), giving a more accurate and less noise-prone readout of the biological response to each media formulation. As such, we used the end-point growth metric of 'percentage change in cell number'. To normalise any small differences in cell numbers seeded, and to enable clear visualisation of the different cost/growth performance for each media mix, the data representation was distributed across one logarithmic (Fig. 2A) and two linear (Fig. 2B, C) plots.

13 mixes screening procedure reduced cell numbers by 70%-100% from the number seeded (Fig. 2C). For the 7 mixes in the Fig. 2B plot, performance ranged from very little change in cell number to approaching 50% decrease. Further investigation, outside the scope of this current study, may be warranted into the effect of adapting cells to grown in mixes G, F and Y, given their sub-£50/L cost profiles (Fig. 2B).

Of the complete media products, AIM-V (Mix AC) and M199 (Mix H) dramatically reduced cell numbers (Fig. 2C), BHK21 (mix G) also reduced cell numbers, but to lesser degree (Fig. 2B), while Hams F12 (Mix N) and IMDM (Mix I) supported cell growth (Fig. 2A). Of the base media, three mixes incorporating Iscoves (K, L M) and two mixes (A and D) with Fishers media supported cell growth (Fig. 2A). None of the other base media (DMEM, Swims, Williams) supported cell growth except Ames, for which one mix (AB) supported growth at the highest level observed of all screened mixes (Fig. 2A). For each of the four serum alternatives, six different mixes were tested. Two mixes supported growth for Ultroser (D and K), AOF-ITS (A and L) and B27 (AB and X), while one Knockout mix (M) supported growth (Fig. 2A).

Figure 2 includes the cost/growth profile of 20% v/v FCS DMEM (plot A, data point labelled '20'). Notably, only four media mixes (AB, X, AA and W) had a greater volumetric cost than 20% v/v FCS DMEM, while all other mixes had a lower cost. None of the media mixes with greater cost than 20% v/v FCS DMEM supported higher levels of growth than 20% v/v FCS DMEM.

### Cost-performance analysis identifies Mix D as a promising serum-free media with 33% lower volumetric cost than standard media

With respect to selecting a media mix for further investigation, we considered the cost dimension to be moot for the mixes that did not support growth in the context of our binary screen (Fig. 2B, C), shortlisting only those that supported an increase in cell growth (Fig. 2A). Of those mixes, seven had lower volumetric cost than 20% v/v FCS DMEM. Amongst those seven, Mix D—Fishers base medium containing 2% v/v Ultroser G serum alternative, supported the highest level of cell growth, while representing a 33% volumetric cost reduction compared to 20% v/v FCS DMEM.

### Lactate production and growth dependence of Ultroser G in Mix D

The Ultroser G serum alternative accounts for 94% of the £97.84/L volumetric cost of Mix D (Table 1), so we next determined whether the level of cell growth support provided by Mix D would be preserved at lower concentrations of Ultroser G. Reducing the volume per L of Ultroser G by 25% and 50% could potentially reduce the overall volumetric cost of Mix D to £74.87/L and £51.92/L respectively. However, endpoint growth levels achieved by both these reductions were markedly reduced compared to unchanged Mix D (Fig. 3A).

An alternative to reducing Ultroser G concentration may be to add a relatively low cost supplement, such as a carbon source, to Mix D. To gain insight into this, we analysed Mix D and 20% v/v FCS DMEM using a CuBiAN Bioanalyser which revealed that 20% v/v FCS DMEM contained four times more glucose than Mix D (Fig. 3, compare panels B and C). This indicated that there may be headspace to supplement Mix D with additional glucose, a relatively low cost addition, in order to increase growth performance, within a broader multi-factor optimisation framework—one that could simultaneously assess glucose alongside other formulation variables (e.g. amino acid concentration, osmolarity) to capture potential interaction effects. We flag this as a priority direction for future media optimisation work, outside the scope of the current study.

The profile of molar glucose per cell was very similar between cells cultivated in 20% v/v FCS DMEM and Mix D throughout the 12 day cutlivation, indicating similar levels of glucose consumption per cell (Fig. 3D). However, despite these similar levels of glucose utilisation, levels of lactate per cell were approximately tenfold lower for cells grown in Mix D for every post-seed analysis except day 9 (Fig. 3E). Lactate accumulation can acidify culture medium, often requiring more frequent media changes or additional buffering capacity, both of which can increase operational complexity and cost. For animal cell lines with long track records of industrial use, such as Chinese hamster ovary (CHO) cells, lactate accumulation can be associated with cellular growth, and lactate consumption with transgene expression (Jain et al. 2025). In emerging sectors such as cultivated meat, optimising metrics such as cell biomass accumulation and cell differentiation at scale remain ongoing areas of investigation, so objectives for lactate consumption or accumulation may vary depending on objectives such as biomass accumulation and how differentiation might impact flavour and mouthfeel (Goswami et al. 2025).

### Maximal Sol8 growth in Mix D invariant to clone isolation or media adaptation

In bioprocess settings, genetic drift in mammalian cells has been observed after a period of multiple passages in a new condition, such as a change of growth medium. This adaptation can arise from mechanisms including transcriptional reprogramming, metabolic rewiring, and selection of pre-existing genetic variants (Huhn et al. 2022). We cultivated Sol8 cells over 3 passages in Mix D (Fig. 4A) to obtained what we referred to as the Mix D-adpated cell line, 'Sol8_D'. When cultivated in Mix D, the Sol8_D cell line and Sol8 cells that had been transferred from 20% v/v FCS DMEM in one step, had comparable growth profiles (Fig. 4A) and showed no statistically significant difference in their cell-specific growth rate (Fig. 4B).

The absence of such adaptation effects despite multiple passages indicates that the growth limitation cannot be overcome through transcriptional or metabolic reprogramming alone, strongly suggesting an absolute nutritional limitation or cytotoxic effect. As with the relatively low level of glucose in Mix D (Fig. 3B, C), this suggests some headspaces exists to improve the Mix D formulation.

Clonal variation is a well-documented phenomenon in mammalian cell culture, where individual clones can exhibit substantial differences in proliferation rates, metabolic profiles, and protein productivity even when derived from the same parental line (Lee et al. 2018). We isolated in Mix D four clonally-derived populations ('clones') from the Sol8_D cell line by limiting dilution. We then assessed their growth performance in Mix D (Fig. 4C). Although the number of comparator clones was small, their growth profiles were very similar to each other and to the original parental, polyclonal, cell line (Fig. 4A). The absence of clonal variation in this context indicates that the level of growth supported by Mix D is defined by fundamental metabolic or signalling pathways that are uniformly constrained across the Sol8 population.

### Mix D compatible with pig and mouse fat and muscles cells

We next tested if Mix D could support growth at levels comparable to standard media for three further cell lines, also after a one-step media swap procedure, with no adaptation or clone isolation (Fig. 5A–C). In the emerging field of cultivated meat, it is anticipated that cell types that compose muscle and fatty tissues in their native animal body plan contexts may be critical to the development of commercially viable products (Post and Doğan 2025). These would include myogenic cells for muscle and adipocytes for fat.

From a bioprocess development perspective, demonstrating that a single serum-free medium formulation can support multiple cell types could potentially offer substantial economic and operational advantages. Multi-lineage co-culture approaches, where myogenic and adipogenic cells are expanded simultaneously or sequentially in shared bioreactors, represent a promising route toward cost-effective production. Such approaches may eliminate the need for separate culture vessels, reducing medium preparation complexity, and minimizing contamination risk during transfers between differentiation steps (Bomkamp et al. 2022). However, realizing these advantages requires that the growth medium accommodates the disparate nutritional and signalling requirements of constituent cell types.

We first determined the ability of Mix D to support the growth of cells of the murine pre-adipocyte cell line, 3T3 L1 (Green and Kehinde 1974), which grow in a fibroblast morphology from which they can be induced to differentiate into adipocytes. 3T3 L1 cells were cultivated in their standard media, 10% v/v FBS DMEM, before direct, one-step transfer to Mix D prior to seeding. Subsequent growth over 12 days, showed that Mix D successfully supported cell growth on a level comparable to 10% v/v FBS DMEM for the first 6 days of cultivation, but with tenfold less cells at the endpoint (Fig. 5A).

Pork is the most popular meat globally, for human consumption (Drewnowski 2024) and so represents what is likely to be a strategic priority for the emerging cultivated meat sector. As such we next tested whether Mix D could support growth of the porcine muscle cell line, pMYOSC and the porcine adipose-derived stem cell line, pADSC.

Cells of the pMYOSC cell line were grown in their standard media, 20% v/v FBS DMEM, before the same Mix D transfer and seeding procedure (Fig. 5B). Subsequent growth showed that Mix D successfully supported cell growth to the same endpoint level as 20% v/v FBS DMEM, but with an initial dip in cell numbers over the first six days. For the equivalent procedure, pADSC cells were one-step transferred from 10% v/v FBS DMEM to Mix D (Fig. 5C). Growth was supported at levels comparable to 10% v/v FBS DMEM for 9 days, but with approximately 50% less cells at endpoint.

### Cultivation in Mix D compatible with differentiation of mouse cells into adipocytes

Where terminally differentiated cell types, such as adipocytes and myocytes, may be required for final product quality (Bodiou et al. 2020), retaining the capacity of cells to differentiate during upstream bioprocess, steps such as cultivation, can be critical. As such we next sought to test the ability of Mix D to support growth of cells in undifferentiated state, whilst preserving their capacity to differentiate. We cultivated 3T3_L1 cells in Mix D over 3 passages (as in Fig. 5A), and considered the cells at the end of this procedure as having been adapted to growth in Mix D, designating them as '3T3_D'. We then cultivated 3T3 L1 cells in 10% v/v FBS DMEM and 3T3_D cells in Mix D for 7 days. On day one post-seeding, both cell types showed typical adherent cell morphology and zero detectable staining with the reagent, Oil Red-O, a compound shown by Green and Kehinde (1974) to specifically stain lipid vesicles within adipocytes (Fig. 5D, images 1–4). At Day 7 each media type was replaced with fresh media in both cases, and still no morphplogy change or Oil Red-O binding was observed 7 further days after this change (Fig. 5D, images 5–8). However, for a duplicate seeding, at Day 7 both media were instead swapped for StemPro™ Adipogenic Differentiation media (ADM). 7 days after transfer to ADM, both 3T3 L1 and 3T3_D cells showed an aggregated morphology and enhanced Oil Red-O staining characteristic of adipocyte differentiation (Fig. 5D, images 9–12).

## Discussion

For the majority of commecial entities in emerging fields such as cultivated meat, finite resource budgets are an unavoidable reality. A finite budget scales inversely between the breadth of conditions tested and the number of experimental replicates that are performed per condition. To mirror this commercial reality, we firstly performed an n = 1 screen to detetermine the binary property of 'does/does not suport growth'—allowing us to test 29 media mixtures at the same costs as approximately 10 mixtures at n = 3.

This staged approach is common practice for high-throughput biological screening in commercial settings (Macarron et al. 2011; Butkiewicz et al. 2017) where primary screening runs are frequently performed without replication, with confirmatory assays subsequently applied only to the reduced hit set. The same primary/confirmatory structure is also described Goktug et al. (2013), where primary screens are typically single-copy and replication is reserved for the confirmatory stage. For the binary property tested here, of 'does/does not suport growth', we considered that the signal-to-noise ratio at n = 1 was likely to be materially better than for detection of marginal differences in growth profile, and statistically distinct from the experimental noise floor, even without replication.

### Limitations and benefits of a low-replicate screening approach

Inherent to this approach is the fact that we cannot remove the risk of false negatives. In light of this we would emphasize that our observations with respect to the media mixes that were excluded from further investigation after the binary screen (Figs. 1 and 2) should not be considered as definitive, or given overdue weight. Our data were used only to inform the deprioritisation of certain mixes with the understanding that they remained available for re-evaluation at a future time. Our cost-performance mapping (Fig. 2) exemplifies how a repurposing screen approach can quantitatively balance economic and biological criteria.

Such analysis may be especially valuable for translational applications, where incremental cost savings in growth media can significantly reduce total production cost per kilogram of cultivated biomass (Humbird 2021). In future, cultivation at larger (e.g., bioreactor) scale will allow accurate biomass-based quantification to be used to assess media mix performance.

Both excess and scarcity of nutrients can potentially lead to cell death (Wellen and Thompson 2010) so a diverse set of factors may underly why each of the 20 media mixes that suppressed or failed to support growth (Figs. 1 and 2).

For the media mixes that caused the most marked reduction in cell numbers (Fig. 2C), a significant mismatch of nutrient availability, osmolality, pH regulation or growth factor availability are likely explanations (Krampe and Al-Rubeai 2010; Patil et al. 2020). The degree to which some of the media components were finely optimised for specific cell types is also likely to be a factor in their failure to support growth of the Sol8 cell line.

### Sol8 cell growth supported by media mixes costing below £50/L

Media Mixes I, A and L each had a cost base of below £50/L (Table 1), while supporting percentage increase in cell number of 66.4%, 217.5% and 556.2% respectively (Fig. 2A). This combination of low material cost and measurable proliferative benefit is notable given that most cost-competitive formulations in this study did not yield comparable growth gains (Fig. 2B, C), and suggests that sub-£50/L formulations are not inherently limited in performance potential.

These three mixes were not carried forward for further characterisation as we ranked Mix D higher overall, as it supported an increases in cell numbers more then sixfold greater than Mix L (3,206.9%) whilst still having a sub-£100/L cost base. However, we recommend Mixes I, A and L be considered for further characterisation in future work, particularly through (i) replication across independent cell banks or passage numbers to confirm robustness of the growth effect, (ii) extended culture duration to assess whether early growth advantages are sustained or attenuated over time, and (iii) formal cost-per-cell or cost-per-unit-biomass analyses that combine the media cost data presented here with achieved growth rates, to better contextualise their economic advantage relative to higher-cost alternatives.

### Sol8 cell growth supported by Mix D

Within Mix D, the concentration of Ultroser G correlated with ability to support cell growth (Fig. 3A), indicating that its constituents were critical for supporting Sol8 cell growth, in terms of either identity, pH, molality or a combination thereof. The lower metabolic activity Sol8 cells exhibited (Fig. 3) suggests that Mix D promotes proliferation through a distinct metabolic state, potentially involving altered amino acid or lipid utilization. Previous work has shown that serum-free formulations can shift cellular energy metabolism, sometimes resulting in lower basal respiration but sustained proliferation (Kim and Lee 2009).

Cultivation of Sol8 cells in Mix D over 3 passages allowed us to obtain a population of cells, Sol8_D, that we described as having been adapted to Mix D (Fig. 4A, B). When cultivated in Mix D, Sol8_D cells did not exceed or fall below the growth performance of the parental Sol8 population in Mix D, throughout a 12-day cultivation (Fig. 4A) and showed no difference in cell specific growth rate (Fig. 4B). This observation indicated that the growth support provided my Mix D was not dependent on stochastic adaptation or clonal drift.

We used limiting dilution cloning to optain four clonally-derived subpopulations from Sol8_D (Fig. 4C). All four Sol8_D clones matched the growth performance of the parental Sol8 and Sol8_D populations in Mix D (compare Fig. 4A and C). This observation is consistent with a scenario in which Mix D did not exert strong selection pressure on the parental Sol8 cell line, and the cell growth observed (Figs. 1, 3) was not due to emergence of a selected, fast-growing sub-population.

The failure of clone screening or adaptation to result in increased growth performance may also indicate that Mix D lacks certain peptide or steroidal components present in FBS that support myoblast proliferation, and that the metabolic and signalling rewiring necessary to fully compensate for these differences exceeds the adaptive capacity of the cells tested. Nevertheless, maintaining stable proliferation performance across multiple passages and without the need for clone isolation suggests that D provides a sufficiently complete trophic environment to sustain long-term culture without progressive decline.

### Growth of three further cell types supported by Mix D

The immortalized lines tested here (Sol8 and 3T3) are adapted to standard, serum-containing growth media, so are potentially not predictive of the limitations that become apparent with primary cells or cells from agricultural species.

The ability of Mix D to support both cell lines-murine fibroblasts as well as porcine primary myosatellite and porcine adipose-derived stem cells-underscores its potential as a broadly applicable serum alternative (Fig. 5A–C). Cross-species compatibility is a critical requirement for media intended for industrial-scale animal cell culture, where species-specific media development can be cost-prohibitive. Furthermore, the preserved adipogenic differentiation of 3T3 L1 fibroblasts after growth initial growth in Mix D (Fig. 5D) confirms that functional phenotypes are maintained.

While Mix D supported multi-lineage cell types, the kinetics were not uniform across species and lineages. Porcine pMYOSC exhibited an extended lag phase of up to six days before entering exponential growth (Fig. 5B), and both 3T3-L1 mouse and pADSC cells reached significantly lower endpoint cell densities than in standard medium (Fig. 5A and C respectively). These differences likely reflect species- and lineage-specific requirements for attachment factors, growth factor combinations, or metabolic substrates that Mix D does not yet fully satisfy for every cell type tested, as well as possible differences in each line's basal adaptation to serum-reduced or serum-free conditions.

From a bioprocess perspective, a prolonged lag phase directly extends batch duration, and a lower endpoint density reduces volumetric productivity, both of which raise per-unit production costs at industrial scale. We consider these limitations potentially tractable through several complementary strategies. First, lineage-specific formulation adjustments—for example, supplementing Mix D with additional growth factors or lipids known to support pADSC and 3T3-L1 proliferation, or pre-conditioning porcine pMYOSC through a brief adaptation passage before switching to Mix D—could shorten the lag phase and improve final densities. Second, process-level interventions such as fed-batch feeding regimes or partial perfusion could sustain nutrient availability during the extended lag and support higher terminal densities without requiring full medium reformulation. Third, seed train optimization (e.g., staggering inoculation density or extending pre-culture time in standard medium before transition to Mix D) could reduce the practical impact of an initial lag phase on overall batch timelines. We are pursuing lineage-specific formulation refinements and adaptation protocols in ongoing work, and we view resolving these line-dependent kinetics as a priority for translating Mix D from a proof-of-concept multi-lineage medium into a scale-ready platform.

The exact composition of Mix D remains proprietary, limiting mechanistic interpretation. Detailed metabolomic and proteomic analyses could elucidate which components compensate for the absence of FBS-derived growth factors. Additionally, while this study tested several mammalian lines, extending evaluation to a wider selection of primary cells and stem cells would clarify Mix D's suitability for more demanding systems.

Beyond performance benchmarking, future work could explore blending cost-effective serum replacements with targeted growth factor supplements to fine-tune metabolic performance and product yield. The data-driven framework used here could be expanded into a multi-dimensional optimisation pipeline that integrates growth, differentiation, cost, and sustainability metrics.

### Wider context of serum replacement strategies

Sophisticated approaches to identify serum alternatives for animal cell culture can readily be absorbed by research and development budgets in the biomedicine sector. For example, Lin et al. (2019) used metabolomic analysis to identify 28 candidate metabolites associated with improved growth of DF-1 chicken fibroblasts as cell factories for vaccine biosynthesis. These metabolites were consolidated into 11 nutrient groups, screened in 12 experiments to identify the most influential variables, before the three highest-performing groups were further optimised in 15 experiments, increasing cell density by 40.7% and vaccine productivity by 168%.

Cutting edge techniques are also being brought to bear within the emerging cultivated meat commercial sector and in academic research (Quek et al. 2024; Liu et al. 2026; Xie et al. 2026). Skrivergaard et al. (2023) screened 32 combinations of recombinant growth factors and supplements using high-throughput image-based cytometry, followed by statistical optimisation and transcriptomic validation in bovine satellite cells, porcine satellite cells and murine C2C12 myoblasts. This reduced the final formulation to only four essential supplements, containing five proteins (FGF2, fetuin, bovine serum albumin and insulin-transferrin-selenium). These supplements supported proliferation comparable to, or exceeding, serum-containing medium. Although such approaches simplify media composition, the resulting cost savings remain difficult to quantify because recombinant protein production and purification costs vary considerably with manufacturing process and scale.

Biological responses that identify candidate media supplements can also be identified by screening. Messmer et al. (2022) used transcriptomic profiling of bovine satellite cell differentiation to identify receptors induced following serum withdrawal before screening their corresponding ligands, enabling formulation of 10 different chemically defined differentiation media that were then screened for their ability to eliminate the need for serum starvation. Artificial intelligence is now extending these approaches further.

Cosenza et al. (2023) applied Bayesian optimisation to iteratively select the most informative media formulations based on 26 components, identifying serum-free media that increased C2C12 growth by 23% while reducing media cost by 37.5% relative to the control formulation. Similarly, Oey et al. (2026) screened cultivation, recycling and processing conditions for microalgal lysates, enabling a 50% reduction in serum and amino acid supplementation while increasing mammalian cell proliferation by approximately 40%.

More recently, Dolgin et al. (2025) screened 21 microbial lysate variants as inexpensive serum replacements, identifying *Vibrio natriegens* lysate as a low-cost supplement that supported long-term proliferation of immortalised bovine satellite cells and mackerel muscle cells, illustrating the continued transition from purified recombinant proteins towards complex, low-cost biological supplements.

### The challenge and opportunity of proprietary media

The proprietary nature of the commercial media components listed in Table 2 means the exact compositions of these formulations remain undisclosed. Currently, this would constrain the ability to rationally optimize or reformulate these components for cost or process control at scale. Metabolomics approaches, together with complementary analytical approaches such as proteomic or lipidomic profiling of spent and fresh media, offer a potential route to reverse-engineer the functional components driving cell growth and lineage support within these proprietary mixes.

Direct commercial partnership with media producers is also an option, in which formulation details are disclosed under agreement. Unlike biomedical applications, where per-batch volumes are typically small and margins are protected by regulatory exclusivity, cultivated meat production at industrial scale will likely require substantially larger volumes of these formulations. This shift in scale could potentially change the commercial calculus for media producers, with the prospect of high-volume, recurring orders for food-grade applications providing sufficient incentive for producers to disclose or co-develop formulations. Pursuing such partnerships, in parallel with analytical deconvolution efforts, could accelerate the transition from proprietary, cost-opaque formulations to open, scalable, and economically viable media for cultivated meat bioprocessing. We consider both directions important avenues for future work building on the present study.

In this work we identified potential 30% cost reduction with an already extant commercial product, using only relatively low cost equipment to screen 29 different media options directly. We also demonstrated the value of adopting an agnostic approach to evaluating tools and products that already exist within an industry (animal cell tissue culture) with an approximately 75-year track record. We suggest that pragmatic, low-cost approaches, such as those we report here, will have an important role in the development of the cultivated meat industry over time, as it inevitably is faced with cyclical increases and decreases in capital investment.

## Conclusions

This study demonstrates that a repurposing screen of commercial serum alternatives can uncover cost-effective substitutes for FBS that sustain mammalian cell growth and differentiation. Among 29 tested formulations, four exhibited good performance in Sol8 cells, with Mix D emerging as the optimal balance between biological efficacy and cost. Despite reduced metabolic activity and an inability to improve performance through adaptation, Mix D supported proliferation of both murine and porcine cell types and preserved adipogenic differentiation, all at up to 33% volumetric cost reduction relative to standard, serum-based media.

These findings support the feasibility of transitioning toward serum-free or serum-replacement media for diverse mammalian systems, aligning with the goals of reproducibility, cost reduction, and ethical refinement in cell culture. The identification of Mix D as a viable, economical FBS replacement provides a foundation for scalable animal cell cultivation in research and biomanufacturing.

The findings presented here align with the growing shift toward sustainable and ethically responsible biomanufacturing. Identifying an affordable, reproducible serum replacement advances the "3Rs" principles of animal research (replacement, reduction, refinement) while directly supporting scalable production of cultured meat and regenerative products.

Our agnostic repurposing screening framework could be applied to other culture parameters-such as basal media composition or supplement formulation-to accelerate the discovery of cost-efficient, serum-free systems. By prioritizing empirical, cost-informed selection, the approach demonstrated here provides a practical roadmap toward more reproducible, ethical, and economically viable mammalian cell culture.

## Data Availability

Raw data supporting the findings of this study are available from the corresponding author on request.
